# The Sleeping Giant: Metabolism and Autophagy in the Generation and Survival of Dormant Polyploid Giant Cancer Cells

**DOI:** 10.1002/mco2.70873

**Published:** 2026-09-20

**Authors:** Silvia Guil‐Luna, María Teresa Sánchez‐Montero, Alejandra Díaz‐Chacón, Antonio Rodríguez‐Ariza

**Affiliations:** ^1^ Maimonides Biomedical Research Institute of Cordoba (IMIBIC) Cordoba Spain; ^2^ University of Cordoba Cordoba Spain; ^3^ Reina Sofia University Hospital Cordoba Spain; ^4^ Cancer Network Biomedical Research Centre (CIBERONC) Madrid Spain; ^5^ Department of Anatomy and Comparative Pathology and Toxicology Faculty of Veterinary Medicine of Córdoba University of Córdoba Córdoba Spain; ^6^ Medical Oncology Department Reina Sofía University Hospital Córdoba Spain

**Keywords:** autophagy, endoreplication, metabolism, neosis, PGCCs, tumor dormancy

## Abstract

A major limitation in preventing cancer relapse is our incomplete understanding of therapy resistance and tumor dormancy, including the role of therapy‐induced polyploidy in generating rapidly proliferating progeny that repopulate the tumor. In this context, polyploid giant cancer cells (PGCCs) are a unique subpopulation traditionally considered terminally senescent due to large size, abnormal DNA content, and inability to undergo mitosis. However, compelling evidence shows that PGCCs generate progeny via amitotic mechanisms (neosis), producing highly proliferative, chemo‐resistant tumor‐initiating cells that drive cancer relapse. Although metabolic adaptations and autophagy support PGCC formation and survival, the mechanisms underlying these processes and their contribution to tumor relapse remain poorly understood. This review explores the complex interplay between autophagy and metabolic adaptations in PGCCs, emphasizing their role in PGCC formation, long‐term survival and dormancy, and in the generation of amitotic progeny that drive tumor regrowth and cancer relapse. Mechanistic insights into how autophagy and metabolic plasticity enable PGCCs to withstand stress, maintain energy homeostasis, and escape therapy are highlighted. Additionally, we discuss therapeutic approaches targeting PGCCs by disruption of autophagy and metabolic pathways, evaluating their potential to impair progeny formation, overcome therapy resistance, and prevent cancer recurrence.

## Graphical abstract

Polyploid giant cancer cells (PGCCs) arise in response to stress and play a critical role in therapy resistance and tumor dormancy. These cells can evade conventional treatments and later contribute to cancer relapse by generating progeny through nonmitotic, amitotic mechanisms. Their formation, survival, and ability to produce offspring are supported by a dynamic interplay between autophagy and metabolic reprogramming. Targeting this autophagy–metabolism axis in PGCCs may offer a novel strategy to prevent cancer recurrence

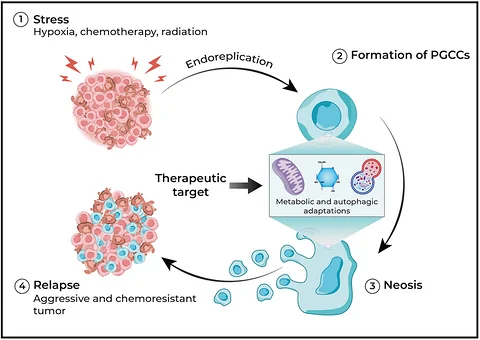

## 1 Introduction

A major obstacle in cancer treatment is the rapid development of resistance to drug therapies by many cancer types. Additionally, some cancers exhibit an encouraging initial clinical response, leading to the patient being considered free of cancer. However, a considerable number of these cancers actually become dormant and subsequently relapse or metastasize after a prolonged period, spanning months or even years [1]. This recurrence is always devastating, but when the cancer lies dormant for an extended period before relapsing, the psychological impact on patients is even worse [2]. Yet, despite intensive research, we are still far from having the ability to effectively target dormant cancer cells to prevent disease relapse in clinical settings. This is due, at least in part, to our limited understanding of regulated proliferation arrest in tumor cells. This process, which plays a crucial role in organismal development, is also believed to be pivotal in tumorigenesis and cancer progression, particularly in terms of therapy resistance, tumor dormancy, and delayed recurrence [3]. Historically, the concept of tumor dormancy has evolved from early clinical observations of late relapse to a complex and dynamic field integrating cell biology, microenvironmental regulation, and therapy response, reflecting its growing relevance in modern oncology. Currently, the field of tumor dormancy is confounded by a proliferation of terms, many of which are vaguely defined and often used interchangeably. Descriptions such as “quiescent,” “slow‐cycling,” “drug‐tolerant persister,” “reversibly senescent,” and “cancer stem cell‐like” are frequently applied to what are broadly referred to as “dormant” cancer cells. These overlapping terms reflect the biological resemblance between dormant cancer cells and other nonproliferating cell types, particularly those in quiescent or senescent states [3]. Thus, cellular dormancy is commonly characterized by two distinct growth arrest mechanisms: quiescence and senescence. Quiescence represents a nonproliferative or slow‐cycling state in which cells exhibit a reversible growth arrest [4]. On the other hand, senescence refers to a state of cell cycle arrest that is predominantly irreversible [5, 6]. Consequently, whether the cancer cells became dormant via quiescence or senescence directly impacts their potential for reactivation and subsequent tumor relapse. Thus, senescence has usually been regarded as a permanent and irreversible state, leading to a terminal fate. However, this traditional view has recently been questioned due to emerging research indicating that therapy‐induced senescent cancer cells have the capability to evade the imposed cell cycle arrest and re‐enter cell cycle progression [7, 8, 9, 10, 11, 12]. Notably, in vitro studies suggest that polyploidy may enable senescent tumor cells to escape their growth‐arrested state, thereby providing a potential mechanism by which these cells evade senescence and resist the cytotoxic effects of anticancer therapies [13, 14].

In this regard, polyploid giant cancer cells (PGCCs) are a distinct population of cancer cells that have long been regarded as nondividing senescent cells due to their inability to undergo mitosis [15]. PGCCs are characterized by their large size and increased DNA content due to abnormal DNA replication and chromosomal duplication [15, 16, 17, 18]. These cells can arise spontaneously within tumors or can be induced by different stressors, such as hypoxia [19, 20], chemotherapy [15, 21], or radiation therapy [22]. It is noteworthy that PGCCs are major contributors to the cellular atypia that pathologists utilize to determine the malignant grade of tumors. This raises an important question regarding how these proliferation‐arrested (senescent) cancer cells can potentially impact malignancy. Recent research supports that PGCCs represent a distinct and important subpopulation of dormant cancer cells that contribute to tumor relapse and therapy resistance by surviving treatment‐induced stress, entering a reversible growth‐arrested state, and later reactivating to repopulate the tumor. In this context, growing evidence indicates that PGCCs can generate progeny cells via amitotic mechanisms, and that these daughter cells display robust mitotic activity, enhanced chemoresistance, and possess tumor‐initiating capabilities [15, 17, 18, 19, 20, 23, 24].

Evidence that polyploid or multinucleated cells can generate viable, proliferative progeny through noncanonical division mechanisms predates much of the recent work on PGCCs. Experimental studies in human cell systems demonstrated that polyploid cells, including near‐senescent or multinucleated populations, can undergo genome reduction through processes such as nuclear budding, karyoplast formation, and reductional divisions that bypass conventional mitosis, giving rise to mononuclear descendants with renewed proliferative capacity. These pioneering observations provided early mechanistic support for depolyploidization as an active, regulated process rather than a terminal aberration, and established amitotic progeny generation as a plausible route for cellular re‐entry into the cell cycle following polyploidization, particularly under stress conditions or growth arrest [25, 26, 27, 28, 29, 30].

Despite recent advances in PGCC research, a comprehensive synthesis of the mechanisms governing PGCC biology, particularly in relation to their metabolic and survival adaptations, remains lacking, highlighting the need for an updated and integrative review. One important aspect of PGCCs is that they must undergo metabolic adaptations to meet their energy demands and ensure their survival. On the other hand, autophagy is a key cellular process that enables cells to maintain their health and functionality by removing damaged or unnecessary components and providing nutrients by breaking down their building blocks [31]. This process has emerged as a critical process in cancer biology, influencing tumor growth, metastasis, and therapy resistance [32, 33]. Moreover, in the context of PGCCs, autophagy appears to be intricately linked to their metabolic adaptations. Given this close interconnection, understanding how metabolic rewiring and autophagy cooperatively regulate PGCC behavior across different stages of their life cycle is essential to fully elucidate their role in cancer progression and therapeutic resistance.

Therefore, this review aims to summarize recent research on the interplay between autophagy and metabolic adaptations in PGCCs, shedding light on their implications for therapy resistance and tumor dormancy. Specifically, we will highlight how these interconnected processes contribute to PGCC formation, maintenance during long‐term dormancy, and the generation of aggressive progeny cells. We will begin by reviewing the concept of PGCCs, with a focus on their formation mechanisms and the underlying biological processes. Next, we will explore the intricate roles of metabolism and autophagy in cancer, highlighting their involvement in the formation of PGCCs, their adaptation for surviving stress, and their ability to generate progeny that repopulate the tumor with more aggressive and resistant cells. Lastly, we will discuss the therapeutic implications arising from the intersection of autophagy and metabolism in PGCCs, exploring the potential to effectively target these dormant cells as a strategy to combat relapse in cancer patients.

## 2 Origin, Induction, and Formation of PGCCs

**FIGURE 1:**
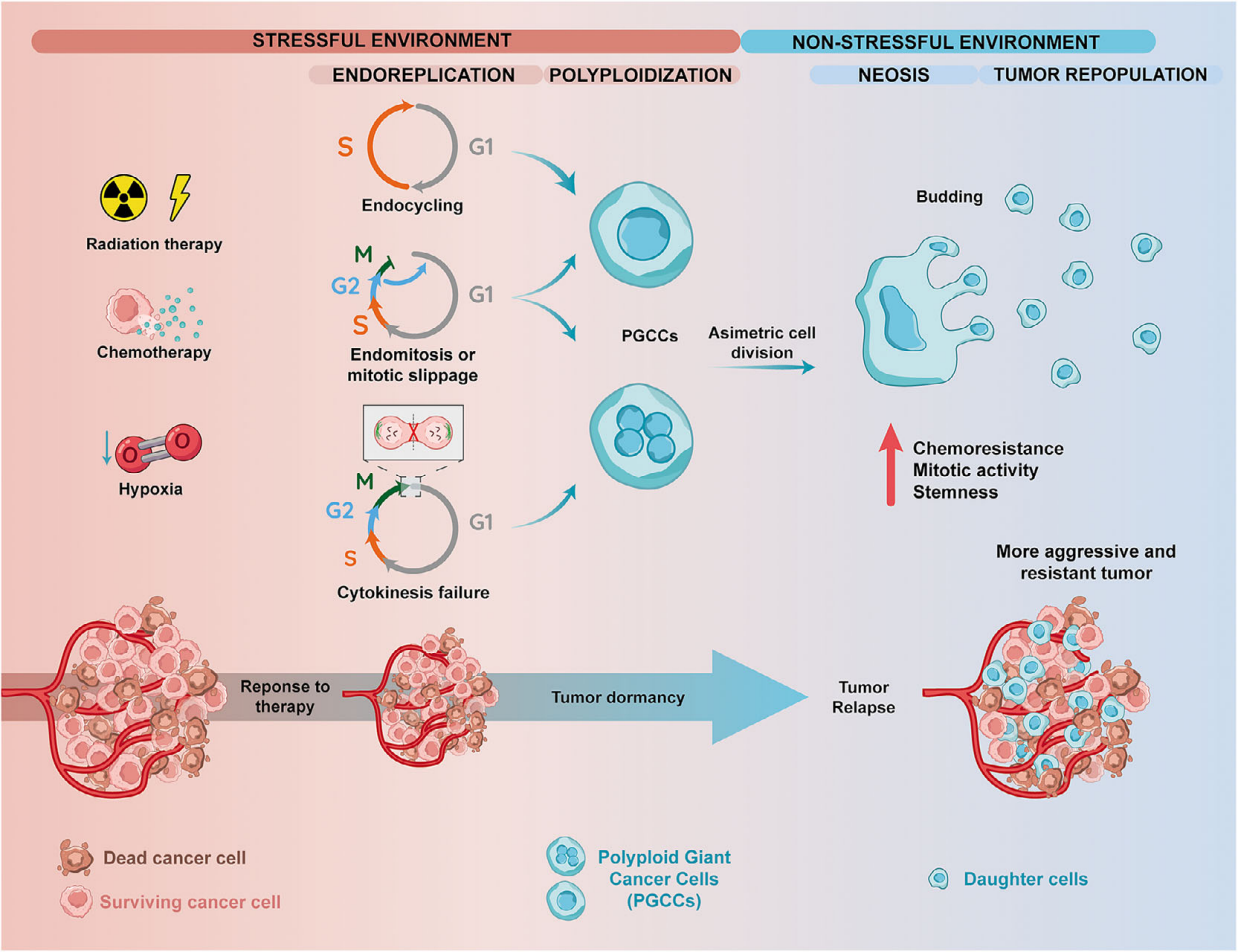
Origin and formation of polyploid giant cancer cells (PGCCs). Schematic representation of the three main endoreplication pathways leading to polyploidization: endocycling, endomitosis/mitotic slippage, and cytokinesis failure. Various stress factors, such as radiation therapy, chemotherapy, or hypoxia, trigger endoreplication pathways, leading to the formation of PGCCs. Under persistent stress, PGCCs enter a dormant state, but upon stress removal, they can undergo neosis, producing smaller, aneuploid daughter cells that re‐enter the cell cycle. This process contributes to tumor relapse, therapy resistance, and increased cancer aggressiveness. See text for details.

Polyploid cells represent a fundamental adaptive strategy across organisms, balancing genomic expansion with cellular function. In this section, we provide an overview of the mechanisms leading to PGCC formation, highlighting both physiological and pathological contexts. Polyploid cells are formed in numerous organisms and organs through a process called endoreplication (Figure 1). This mechanism involves repeated rounds of DNA replication without cell division, resulting in abnormal cell size and multiple nuclei or a single giant nucleus containing multiple complete sets of chromosomes [34, 35]. Polyploidization is a widespread phenomenon in nature and represents a highly effective strategy for tissue development, growth, and repair [36]. For instance, megakaryocytes, which are specialized cells involved in platelet production, undergo polyploidization during their maturation process [37]. Similarly, trophoblast cells in the placenta of some mammals, such as mice and rabbits, undergo multiple rounds of endoreplication, generating polyploid giant cells that play a vital role in creating the tissue barrier between the mother and fetus [38]. Moreover, polyploidization is known to play a pivotal role during tissue regeneration in organs such as the heart, liver, epidermis, and intestine [39]. In the context of cancer, this physiological process is hijacked and reprogrammed, giving rise to PGCCs, a subpopulation of tumor cells characterized by increased genomic content, cellular plasticity, and enhanced survival capacity under stress conditions [40, 41].

### 2.1 Stress‐Induced Triggers of PGCC Generation

There is no unanimous consensus among authors regarding the precise definition of endoreplication. Moreover, the diverse range of mechanisms involved in endoreplication often leads to confusing nomenclature [35, 42]. Nevertheless, several pathways of endoreplication have been described as involved in the generation of PGCCs, including endocycling, endomitosis, or mitotic slippage and cytokinesis failure (Figure 1). Endocycling encompasses alternating S and G phases of the cell cycle without any characteristic features of mitosis [43]. In contrast, cells undergoing endomitosis or mitotic slippage engage in an ineffective attempt at mitosis, which fails to culminate in cell division, and subsequently re‐enter the S phase [44]. Finally, cytokinesis failure, which refers to the inability of a cell to complete the final stage of cell division, is another mechanism contributing to the observed ploidy changes in tumors [45].

A wide variety of stressors have been reported to induce PGCC generation, including DNA damage caused by chemotherapeutic agents, mitotic stress induced by microtubule‐targeting drugs, hypoxia, oxidative stress, radiation, and metabolic perturbations. These conditions frequently result in cell‐cycle arrest and activation of survival pathways that favor polyploidization instead of apoptosis [46, 47]. The specific factors determining why a cell undergoes certain endoreplication pathways, rather than others, remain unknown. However, current evidence suggests that the type and intensity of stressors, as well as tumor‐specific genetic contexts, play a critical role in defining the pathway leading to PGCC formation [16, 41].

### 2.2 Cell Cycle Dysregulation and Escape From Mitotic Catastrophe

Mechanistically, PGCC formation is closely linked to escape from mitotic catastrophe, a form of cell death triggered by defective mitosis. Under conditions of prolonged mitotic arrest, such as those induced by anti‐microtubule drugs, cancer cells may bypass mitotic checkpoints through mitotic slippage, allowing them to survive despite severe chromosomal abnormalities. This ability to evade mitotic catastrophe and re‐enter the cell cycle in a polyploid state represents a critical survival strategy that contributes to tumor persistence and therapeutic resistance [48].

Cell cycle progression through each phase is regulated by cyclin‐dependent kinases (CDKs) and their partner cyclins. However, abnormal CDK activity can lead to deregulation of cell cycle control, which is a common characteristic in many types of cancer [49]. In this regard, switching from mitotic cell cycles to endoreplication involves the selective loss of CDKs activity [50] and, specifically, CDK1 appears to play a crucial role in determining whether cells undergo endocycle or endomitosis [17].

For instance, endocycling was found as the predominant strategy to evade mitotic catastrophe in prostate cancer cells following treatment with the DNA‐damaging agent cisplatin, thereby leading to mononucleated PGCCs formation [51]. On the contrary, tumor cells treated with anti‐microtubule drugs commonly experience a prolonged arrest in pro‐metaphase, having the ability to undergo endomitosis or mitotic slippage, and progressing into the subsequent G1 phase as multinucleated PGCCs with 4n, 8n, or 16n genomes [52, 53]. However, in certain instances, both endocycling and endomitosis can occur concurrently, leading to the formation of mononucleated or multinucleated PGCCs. This phenomenon has been observed in ovarian cancer cells following treatment with the microtubule targeting agent paclitaxel [43].

### 2.3 Genomic Instability and Chromatin Remodeling in PGCC Formation

A hallmark of PGCC formation is the accumulation of genomic instability, which arises as a direct consequence of aberrant mitosis and polyploidization [54, 55]. The increase in genomic content predisposes cells to chromosomal missegregation, DNA damage accumulation, and large‐scale structural alterations, ultimately generating highly heterogeneous and aneuploid progeny following depolyploidization. This genomic instability is not merely a by‐product of defective mitosis but a dynamic process that fuels tumor evolution [55, 56].

In addition to genetic alterations, PGCCs undergo extensive chromatin remodeling and epigenetic reprogramming, which are essential for their adaptive behavior. These changes include modifications in histone marks, DNA methylation patterns, and chromatin accessibility, enabling the activation of transcriptional programs associated with stemness [57], stress resistance [58, 59], and survival [41, 47]. At the chromatin level, PGCCs and related polyploid cancer states exhibit profound transcriptional reprogramming characterized by the upregulation of embryonic, stemness‐associated, and evolutionarily conserved gene networks, suggesting a partial dedifferentiation process driven by epigenetic plasticity [60]. Importantly, chromatin remodeling in PGCCs is tightly linked to stress‐induced signaling pathways and the DNA damage response [61, 62]. Epigenetic regulators, including histone‐modifying enzymes and chromatin remodelers, are activated in response to genotoxic stress, facilitating chromatin relaxation and enabling DNA repair while simultaneously promoting survival and adaptive transcriptional programs [63]. Collectively, these findings indicate that PGCCs exploit epigenetic plasticity as a central adaptive mechanism, coupling genomic instability with reversible chromatin states to generate highly heterogeneous and therapy‐resistant progeny.

### 2.4 Morphological, Functional, and Molecular Features of PGCCs

A key feature of PGCCs is their distinctive morphology, characterized by markedly enlarged cell size and the presence of either a single giant nucleus or multiple nuclei [64]. These structural features reflect their polyploid nature and are often accompanied by increased cytoplasmic volume and irregular nuclear architecture [41, 65]. A defining functional property of PGCCs is their ability to remain dormant and resistant during periods of stress. Once these stressful conditions subside, PGCCs undergo a type of asymmetric cell division that efficiently generates a progeny of smaller daughter cells. This mode of division, known as neosis (Figure 1), involves a reduction in genetic material, leading to depolyploidization and cell downsizing, ultimately producing aneuploid cells that re‐enter the mitotic cycle [66]. The robust proliferative capacity of PGCC‐derived cells may significantly contribute to the resilience and aggressiveness of tumors. This progeny possesses reproductive potential, enabling the repopulation of the tumor with cells that are often more aggressive and resistant than their progenitors. At the molecular level, PGCCs and their progeny frequently display activation of stemness‐associated pathways and share characteristics with cancer stem cells, including self‐renewal capacity and the ability to generate heterogeneous progeny [15, 41].

In summary, PGCC formation results from stress‐induced endoreplication, cell cycle dysregulation, and extensive genomic and chromatin remodeling, leading to distinctive morphological, functional, and molecular features that enable survival, dormancy, and progeny generation. These adaptive processes are closely supported by metabolic rewiring and autophagy, which play a central role in sustaining PGCC viability, stress resilience, and tumor‐repopulating capacity.

## 3 Metabolism and Autophagy in Cancer

Cancer progression and survival rely on tightly coordinated metabolic and autophagic adaptations. These processes provide the energy, building blocks, and quality control mechanisms necessary for cancer cells to withstand stress, proliferate, and acquire resistance traits. Understanding their interplay is essential for elucidating the mechanisms that enable tumor cells, including PGCCs, to survive and adapt under adverse conditions.

### 3.1 Overview of Metabolic Rewiring and Autophagy in Cancer

Cancer cells acquire distinct metabolic traits during oncogenesis and tumor progression that sustain their uncontrolled growth, enhance their invasive and immune‐evasion capabilities, and enable them to adapt to their environment for survival. These changes are now widely recognized as a fundamental feature of malignant transformation [67]. A major metabolic shift in cancer cells is their enhanced dependence on glucose for energy, a process known as the Warburg effect. First identified by Otto Warburg in the 1920s [68], this phenomenon is characterized by increased glucose uptake and its conversion to lactate, even when oxygen is available. While less efficient than oxidative phosphorylation in terms of energy production, aerobic glycolysis allows cancer cells to divert resources into anabolic pathways, promoting the synthesis of lipids, proteins, and nucleotides essential for cell growth and division. In addition to the Warburg effect, cancer cells alter other metabolic pathways, including fatty acid synthesis [69], amino acid metabolism [70], and the tricarboxylic acid cycle [71], fueling growth and significantly affecting the tumor microenvironment (TME) [72]. For instance, the increased glutaminolysis and glucose consumption by cancer cells deplete the TME of essential nutrients like glutamine and glucose while generating lactate, which weakens T‐cell function and impairs the immune response, allowing tumors to evade immune defenses [73, 74].

Metabolism and autophagy are intricately connected processes that play vital roles in cellular health and function [75, 76]. Metabolism provides the energy and building blocks necessary for cellular activities, while autophagy serves as a quality‐control mechanism, recycling cellular components, including proteins, organelles, and other cellular structures that are damaged, dysfunctional, or no longer needed [77]. The autophagic process involves several key stages, beginning with initiation, which triggers the nucleation of an isolation membrane that forms a cup‐shaped structure called the phagophore, responsible for engulfing damaged organelles and cellular debris. This phagophore then elongates to form a mature autophagosome, which fuses with a lysosome to create an autolysosome. Within the autolysosome, lysosomal enzymes degrade the engulfed material into basic components, such as amino acids and fatty acids. Finally, these breakdown products are released back into the cytoplasm for recycling and utilization, maintaining cellular homeostasis.

Autophagy is highly regulated and relies on the coordinated action of numerous proteins. In mammals, 30 autophagy‐related (ATG) proteins have been identified, with approximately 20 of them playing essential roles in regulating this process [31, 78]. The autophagic process is defined by the sequential and coordinated action of ATG proteins within multiprotein complexes and their interactions with other cell signaling networks. Among these, AMP‐activated protein kinase (AMPK) and the mechanistic target of rapamycin (mTOR) signaling pathways are critically important in the regulation of autophagy. AMPK serves as an energy sensor, activating autophagy in response to low energy levels, thereby promoting cellular homeostasis and survival during metabolic stress. In contrast, mTOR functions as a key inhibitor of autophagy under nutrient‐rich conditions, signaling the cell to conserve resources and suppress the autophagic process. The balance between these two pathways is crucial, as it determines the cell's ability to adapt to changing environmental conditions and maintain cellular health through the modulation of autophagy [79].

### 3.2 Dual Role of Autophagy in Cancer

Autophagy has been found to be dysregulated in various cancer types [80, 81, 82], exhibiting a dual role that either promotes or inhibits tumor growth depending on the disease stage and the specific cellular environment [78, 83]. On one hand, autophagy functions as a tumor suppressor by eliminating damaged or mutated proteins and organelles that could contribute to tumor progression [84]. On the other hand, it can facilitate tumor growth by supplying energy and nutrients to cancer cells, aiding their survival in challenging conditions, such as nutrient deprivation, hypoxia, or during therapy [85]. Defective autophagy resulting from genetic mutations in ATG genes, particularly the haploinsufficient tumor suppressor Beclin 1 (ATG6/BECN1), has been associated with various cancers, including liver, prostate, breast, and ovarian cancers [86, 87]. Additionally, other ATG genes, such as ATG5, ATG2B, ATG7, ATG9B, and ATG12 [83, 88, 89, 90], along with mutations in autophagy regulators such as PIK3CA that activate the mTOR pathway [91], can also disrupt autophagy function, promoting cancer cell growth and survival.

### 3.3 The Interdependent Roles of Autophagy and Metabolism in Cancer Progression

The relationship between metabolism and autophagy in cancer is complex and crucial to various stages of tumor progression. Autophagy not only regulates the metabolism of precancerous cells, aiding their survival under conditions of hypoxia or nutrient deprivation [92, 93], but also supports tumor initiation by facilitating the metabolic processes needed to acquire and maintain cancer stem‐like properties, as evidenced by the promotion of autophagy in glioma and liver cancer stem cells under nutrient‐limited conditions [94, 95]. Autophagy and metabolism interact significantly during the growth and proliferation of cancer cells, with metabolic processes influencing autophagy regulation. For example, glucose scarcity triggers autophagy by activating AMPK and inhibiting mTORC1 [79], while specific glycolytic enzymes, such as glyceraldehyde 3‐phosphate dehydrogenase, phosphoglycerate kinase 1, and hexokinase‐II, can directly modulate autophagy through various mechanisms, including phosphorylation of autophagy‐related proteins [96, 97, 98]. This interplay becomes particularly critical in RAS‐driven cancers, where mutations in RAS increase energy demands and create an autophagy dependency for tumor advancement [99, 100]. The interplay between autophagy and metabolism also significantly contributes to cancer cell metastasis by improving cancer cell invasion and adaptation to new microenvironments while protecting against cell death. For instance, autophagy has been shown to promote glycolysis and metastasis in hepatocellular carcinoma cells [101], and the processes of epithelial‐mesenchymal transition (EMT) and autophagy are closely linked to tumor cell glycolysis, with many stimuli triggering both EMT and autophagy also inducing glycolysis in tumor cells [102].

Finally, the complex interplay between autophagy, metabolism, and cancer is further complicated by stressors that induce autophagy in tumor‐associated stromal cells, influencing the TME through various pathways. While the TME presents challenges, such as low oxygen and nutrient scarcity, tumor cells with metabolic reprogramming can adapt and thrive, with autophagy enhancing the metabolic crosstalk between tumor and stromal cells to promote tumor development [103].

In summary, the dynamic interplay between metabolism and autophagy governs key aspects of cancer cell survival, stress adaptation, and proliferation. This interplay is particularly crucial for PGCCs, where coordinated metabolic rewiring and autophagic regulation support their formation, long‐term stress resilience, sustained survival, and ability to generate progeny.

## 4 Metabolic and Autophagic Drivers of PGCC Formation

The formation of PGCCs is tightly linked to cellular metabolism and autophagy, and this section explores how metabolic rewiring, nutrient sensing, and autophagy coordinate with cell cycle regulation to drive PGCC generation.

### 4.1 Interplay of Metabolism and Cell Cycle Regulation: Relevance for PGCC Formation

**FIGURE 2:**
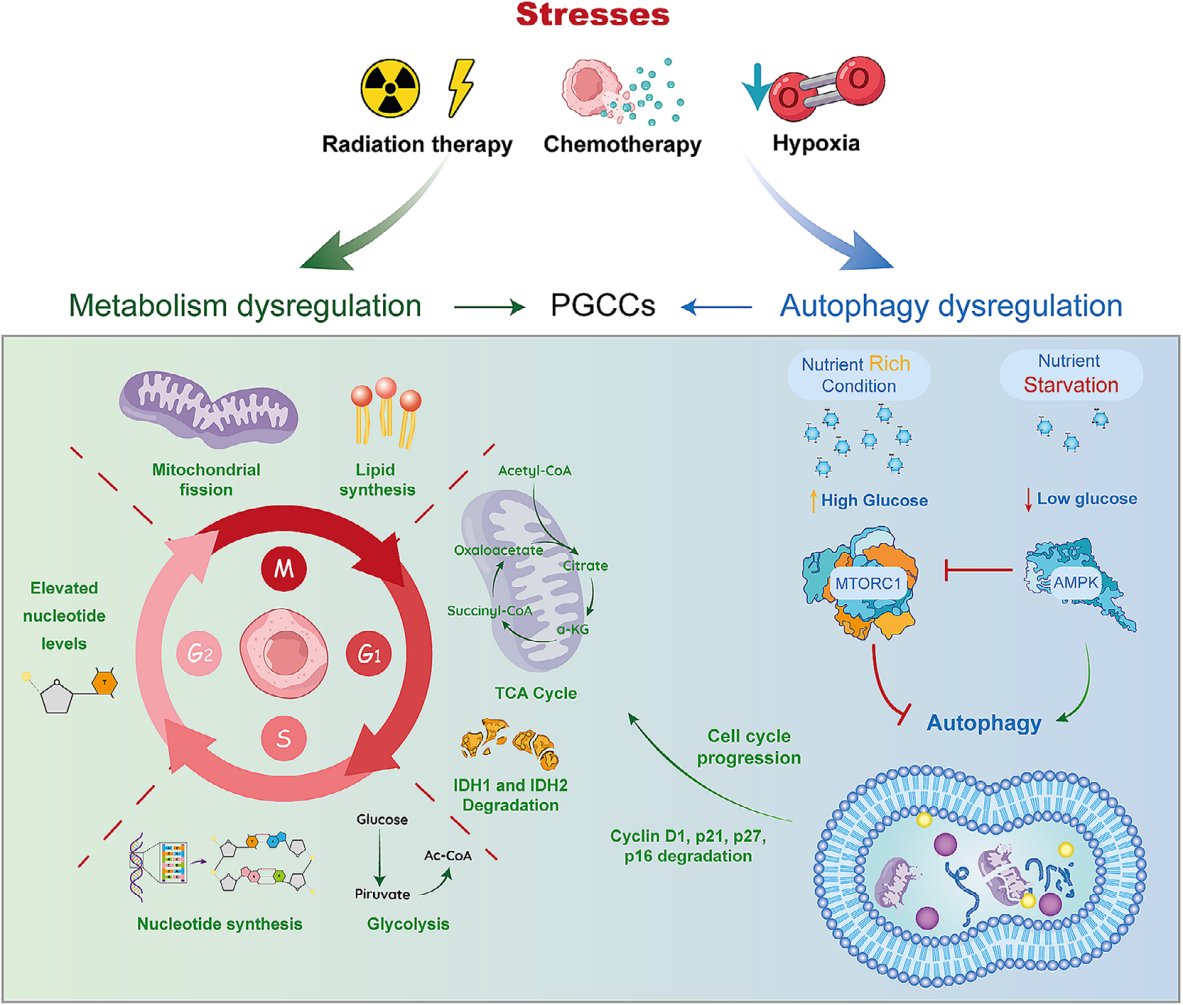
Interplay Between Cell Cycle, Metabolism, and Autophagy: Mechanisms and Dysregulation Leading to PGCCs Formation. Dysregulation of the dynamic interactions between the cell cycle, metabolism, and autophagy can drive the formation of PGCCs. Distinct metabolic pathways are activated during different cell cycle phases, with the tricarboxylic acid (TCA) cycle predominantly active in G1 and glycolysis and nucleotide synthesis upregulated during the S phase. The mechanistic target of rapamycin (mTOR) and AMP‐activated protein kinase (AMPK) pathways serve as central regulators, balancing metabolism and autophagy in response to cellular energy status. Dysregulation in these processes, such as excessive mTOR activation suppressing autophagy or AMPK‐induced metabolic stress, can lead to abnormal cell cycle progression and polyploidization. See text for details.

A crucial factor to consider in the role of metabolism and autophagy in the generation of PGCCs is their complex interplay with cell cycle regulation [104, 105]. Metabolic pathways provide the necessary energy and building blocks required for DNA replication, cell growth, and the synthesis of proteins and other macromolecules needed for cell division. Hence, proper control of cell cycle progression requires tight coordination with metabolic processes (Figure 2), as metabolic activities are adjusted during each phase of the cell cycle to meet the specific demands of that phase [106, 107, 108]. For instance, during the S phase of interphase, nucleotide synthesis pathways are specifically upregulated to meet the demands of DNA replication, resulting in elevated nucleotide levels throughout the S, G2, and M phases [109, 110]. In contrast, during the G1 phase, these pathways are downregulated by the cell cycle inhibitor RB, in line with the lower demand for nucleotide production at that stage of the cycle [111]. Moreover, mammalian cells undergo a bioenergetic metabolic shift during different phases of the cell cycle. Thus, in the G1 phase, cells primarily rely on the tricarboxylic acid (TCA) cycle for energy production but prefer glycolysis in the S phase (Figure 2). This coupling of the cell cycle with bioenergetic metabolism is regulated by the timely degradation of IDH1 and IDH2, which are key enzymes in the TCA cycle [112]. Recent research has also demonstrated that cell cycle regulator proteins influence glucose metabolism through transcriptional regulation and kinase activities [106, 113].

As cells transition from interphase to mitosis, metabolic activities shift toward supporting the physical processes of cell division. Mitosis, in fact, triggers some of the most significant metabolic changes in the cell cycle (Figure 2), including a marked reduction in translation activity and alterations in mitochondrial function, dynamics, and lipid synthesis [104]. These changes affect the cell's energy balance and influence the production of reactive oxygen species (ROS). Energy is required for the rapid remodeling of microtubules during mitosis, which is essential for spindle formation and chromosome segregation [114]. Additionally, mitochondrial fission is crucial during mitosis to ensure proper distribution of mitochondria between the two daughter cells [108], while increased lipid synthesis is required to facilitate the membrane remodeling necessary for cytokinesis [115].

Disruptions to the metabolic changes necessary for proper cell cycle progression can result in cell cycle abnormalities, such as improper DNA replication, failed cell division, or checkpoint bypass. Consequently, metabolic dysregulation that affects processes such as nucleotide synthesis, energy production, or mitochondrial function and dynamics may trigger the formation of PGCCs, contributing to tumorigenesis and cancer progression (Figure 2). In this regard, studies have shown that a reduction in deoxyribonucleotide triphosphate (dNTP) levels contributes to oncogene‐induced senescence (OIS), leading to a stable exit from the cell cycle [116]. This depletion of dNTPs hampers DNA replication, triggering a cellular stress response that enforces permanent cell cycle arrest, a hallmark of OIS [116]. Importantly, multinucleate cells in OIS arise from specific mitotic abnormalities triggered by oncogene signaling, leading to impaired mitosis and subsequent cell cycle arrest [117].

### 4.2 Role of Nutrient Sensing and Autophagy in Cell Cycle Regulation and PGCC Formation

Autophagy is another process that is intricately connected with the cell cycle, with nutrient sensing playing a crucial role in its regulation (Figure 2). Nutrient availability influences both cell cycle progression and autophagic activity, as cells adjust their growth and division based on the availability of essential resources. Nutrient‐sensing pathways, such as mTOR and AMPK [79, 118], coordinate these processes to optimize metabolic and growth responses, maintaining balance between cell proliferation and the recycling of cellular components. mTOR complex 1 (mTORC1) plays a significant role in regulating autophagy, primarily by inhibiting this process during periods of nutrient abundance. When mTORC1 is active, it promotes cell growth and suppresses autophagy [119]. On the other hand, AMPK, which is activated by low energy levels, such as an increased AMP/ATP ratio, nutrient deprivation, and cellular stress, acts as an inhibitor of mTORC1, thereby promoting autophagy [120] (Figure 2). A recent study has shown that low mTORC1 activity during the G1 phase makes cells more sensitive to autophagy induction. Moreover, oscillatory mTORC1 throughout the cell cycle not only influences cell growth and division but also plays a crucial role in determining when and how autophagy is activated in response to cellular needs [121]. Also, elevated ROS levels during mitosis have been proposed to trigger lysosome‐mediated autophagy, which increases amino acid availability and activates mTORC1, thereby promoting cell cycle re‐entry [122, 123]. AMPK also participates in cell cycle regulation, particularly during mitosis, where its phosphorylation by CDK1 is essential for proper chromosome alignment and mitotic progression [124].

Due to its intricate connection with cell cycle regulation, alterations in autophagy may also contribute to the formation of PGCCs (Figure 2). For instance, recent research has shown that chemotherapeutic drugs partially damage mitochondria, leading to reduced ATP levels and activation of autophagy through the AMPK‐mTOR pathway, ultimately promoting the formation of PGCCs [125]. Notably, in this study, the inhibition of autophagy with hydroxychloroquine significantly decreased chemotherapy‐induced PGCC formation. However, another study reported that autophagy inhibition with hydroxychloroquine had no effect on PGCC formation in human ovarian cancer cell lines treated with chemotherapy, although, surprisingly, the mTORC1 inhibitor rapamycin, which is a potent inducer of autophagy, impaired the ability of PGCCs to form colonies [126]. PGCCs may depend on AMPK pathway activation and suppression of mTORC1, promoting autophagy for their formation, whereas dormant polyploid cells require mTORC1 activation and autophagy suppression for their maintenance and propagation (Figure 2). This would explain the strong correlation between mTOR signaling and hallmarks of polyploidy, such as DNA content, augmented cellular size, multinucleation, and asynchronous cell cycle [127].

### 4.3 Autophagy‐Mediated Regulation of Cell Cycle Proteins During PGCC Formation

The role of autophagy in polyploidy is linked to its essential function across various phases of the cell cycle by influencing the activity of cell cycle regulators. Early studies using rapamycin highlighted the critical role of mTOR in cell cycle regulation, particularly through the degradation of cyclin D1 and the upregulation of p27 [128, 129]. Later studies confirmed that cell cycle regulators are direct targets of autophagy during cell cycle progression. Among them, the cyclin‐dependent kinase inhibitors p21 [130], p27 [131], and p16 [132] are degraded through autophagy, which facilitates cell cycle progression (Figure 2). Notably, recent research has identified gene signatures associated with survival in various cancers and highlighted the cell cycle inhibitor p21 as a key player in PGCCs and their early progeny. Significantly, the study demonstrated that blocking p21 expression inhibited PGCC formation [133]. Additionally, autophagy also targets cyclins and CDKs for degradation. The breakdown of these proteins, in contrast, leads to a halt in cell cycle progression. For example, after treating breast cancer cells with DNA‐damaging agents, CDK1 is degraded via the lysosomal pathway, leading to cell cycle arrest to allow time for damage repair [134]. On the other hand, the mTOR inhibitor everolimus induces cell cycle arrest in breast cancer cells by promoting autophagy‐mediated degradation of cyclin D1 [135]. In line with this, we previously reported that the generation of PGCCs in colon cancer cells treated with CoCl_2_, a hypoxia mimic, was associated with decreased cyclin D1 levels, cell cycle arrest, and the expression of a truncated form (p47) of p53 [19]. Notably, p47 is specifically induced during the unfolded protein response (UPR) following endoplasmic reticulum stress [136], and recent research has linked UPR to PGCC formation [137]. Additionally, proper mitotic progression depends on the coordinated degradation of mitotic factors, traditionally attributed to ubiquitin‐mediated, proteasome‐dependent pathways. However, recent findings show that lysosomal impairment prolongs mitotic timing and leads to mitotic errors, demonstrating that mitotic transitions also rely on autophagic lysosome‐dependent degradation [138].

Given the close association between PGCCs and senescent‐like states, it is worth noting that a substantial body of literature has explored the complex relationship between autophagy and therapy‐induced senescence. Although both processes are frequently activated in parallel following cancer treatment, their interdependence remains unresolved and may vary with cellular context. Some studies suggest that autophagy supports the establishment or maintenance of senescence, while others report that its inhibition can enhance senescent phenotypes [139]. In the case of PGCCs, this duality may reflect stage‐specific roles for autophagy, being essential for PGCCs formation, but dispensable for their maintenance and propagation, as discussed above.

Collectively, the coordinated regulation of metabolism and autophagy drives PGCC formation by integrating energy production, nutrient sensing, and cell cycle control. These adaptations not only enable the emergence of polyploid cells under stress but also set the stage for understanding the metabolic and autophagic mechanisms that sustain long‐term PGCC dormancy.

## 5 Metabolic and Autophagic Adaptations Sustaining Long‐Term PGCC Dormancy

PGCCs exhibit unique adaptations in metabolism and autophagy that allow them to survive prolonged periods of stress. This section provides an overview of how metabolic reprogramming, autophagic flux, and signaling pathways coordinate to sustain long‐term PGCC dormancy, supporting their structural integrity, energy homeostasis, and immune evasion.

### 5.1 Metabolic Reprogramming Associated With Increased Cell Size

**FIGURE 3:**
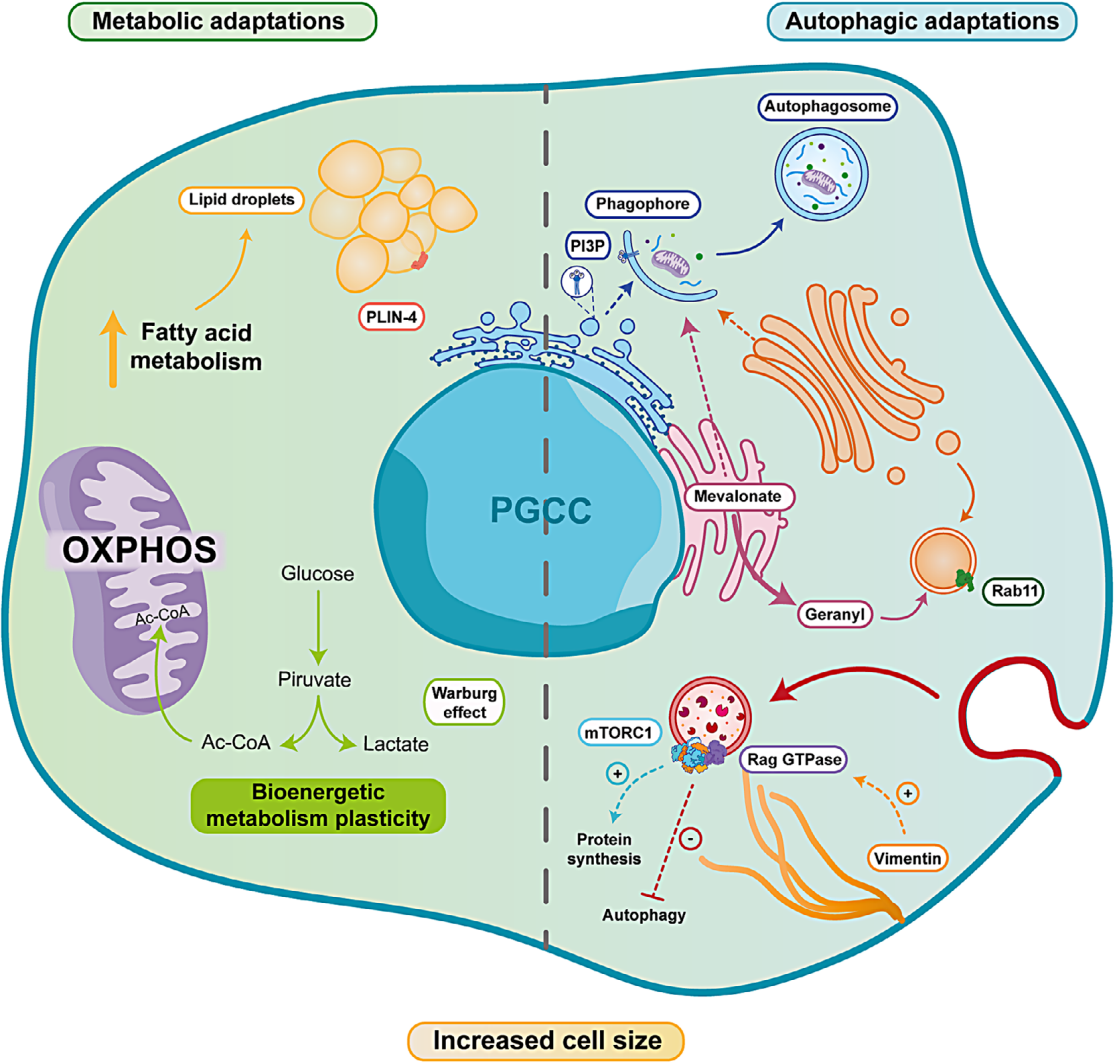
Metabolic and autophagic adaptations in PGCCs. PGCCs present significant metabolic and autophagic adaptations to meet the specific demands of their enlarged size and altered functionality. PGCCs exhibit metabolic plasticity, shifting between glycolysis and oxidative phosphorylation (OXPHOS) depending on the cellular environment. They also rely on fatty acid metabolism and lipid droplets as energy reservoirs. Additionally, molecular mechanisms involving the mevalonate pathway, dynamic PIP3 regulation, and the vimentin network play pivotal roles in PGCC survival by enhancing autophagic adaptations and maintaining their enlarged cellular size. Symbols “+” and “−” denote promotion (activation/induction) and inhibition (repression/suppression), respectively. See text for details.

PGCCs exhibit significant metabolic and autophagic adaptations that contribute to their survival, reprogramming their metabolism to meet the unique demands of their enlarged size and altered functionality (Figure 3). Although the metabolic impact of increased cell size is still largely unclear, a seminal study demonstrated that as hepatocytes enlarge, they undergo significant metabolic reprogramming, characterized by downregulation of mitochondrial gene expression and a marked reduction in mitochondrial oxidative phosphorylation [140]. More recently, studies on polyploid yeast showed a downregulation of several proteins involved in mitochondrial respiration, reinforcing the notion that oxidative phosphorylation declines as cell size increases [141].

Therefore, it is not surprising that a key metabolic adaptation of PGCCs is their increased reliance on glycolysis, even in the presence of oxygen (the Warburg effect), which provides rapid ATP production and biosynthetic precursors for macromolecule synthesis (Figure 3). For instance, acute myeloid leukemia cells treated with aurora kinase inhibitors become polyploid and subsequently enhance glycolysis, indicated by increased glucose uptake and lactate production [142]. In this context, in vitro studies have shown that lactic acidosis can trigger polyploidization as a stress response in transformed cells, primarily through the process of endoreduplication [143]. Moreover, lactylation, which is a recently identified posttranslational modification derived from lactate, has been implicated in the regulation of gene expression and may play a role in tumor dormancy. Thus, in colorectal cancer, the transition to a diapause‐like state has recently been associated with elevated lactate levels that promote histone lactylation, leading to increased expression of ABC transporters and reduced sensitivity of tumor cells to chemotherapy [144]. These findings raise the possibility that lactylation could also influence the formation of PGCCs and their ability to generate progeny, thereby contributing to tumor persistence and relapse. Studies in ovarian PGCCs have reported the upregulation of glycolytic enzymes [145] and reduced mitochondrial membrane potential [146]. Similarly, glioblastoma cells with elevated ploidy, compared with the main tumor population, exhibit enhanced metabolic activity and are especially vulnerable to glycolysis inhibition [147]. On the other hand, recent studies have demonstrated that PGCCs mimic the characteristics of the dedifferentiation program observed in blastomere‐stage embryos [53, 148]. Additionally, the Warburg effect has been proposed to play a critical role in the development of preimplantation embryos [149]. This suggests that PGCCs likely employ a similar Warburg effect for metabolic adaptation, enabling them to survive in harsh conditions [53].

### 5.2 Metabolic Plasticity in PGCCs: Balance Between Glycolysis, Oxidative Phosphorylation, and Lipid Metabolism

PGCCs may also upregulate oxidative phosphorylation when required, allowing for flexible energy production depending on environmental conditions (Figure 3). Hence, glioblastoma PGCCs formed after doxorubicin treatment exhibited a transient induction of oxidative phosphorylation, enabling a fine balance of aerobic and glycolytic ATP production. This metabolic reprogramming facilitated effective ROS management, helping to maintain cellular viability and support survival under the oxidative stress induced by chemotherapy [150]. Notably, in this study, the upregulation of GSK‐3β in doxorubicin‐induced PGCCs and their sensitivity to GSK‐3β inhibition suggested that this kinase may play a role in the metabolic adaptations of PGCCs. In this context, elevated expression of GSK‐3β in tumors has been associated with poor clinical outcomes and prognosis [151]. Moreover, GSK‐3β has been shown to link the metabolic activities of cancer cells with their adaptation to the microenvironment [152].

In another study, doxorubicin‐induced PGCCs in triple‐negative breast cancer cells shifted their metabolism to a higher dependence on fatty acids and oxidative phosphorylation [153]. These PGCCs were, in addition, characterized by the presence of small mitochondria in the cytoplasm as well as a marked enrichment of lipid droplets. Notably, the study identified the enhanced dependence of PGCCs on the lipid droplet‐coating protein perilipin 4 (PLIN4) as a metabolic vulnerability. Additionally, PLIN4 was upregulated in both doxorubicin‐resistant cells and clinical chemo‐resistant tumors treated with neoadjuvant doxorubicin‐based chemotherapy [153]. Similarly, cisplatin‐induced PGCCs in bladder cancer cells exhibited a higher density of mitochondria and an increased number of lipid droplets [21]. Lipid droplets likely function both as energy reserves and protect against lipotoxicity, aiding PGCCs in surviving under stress. The accumulation of lipid droplets may act as storage reservoirs, providing a sustained energy source that enables PGCCs to endure prolonged periods of stress and maintain their survival during harsh conditions (Figure 3). On the other hand, excessive levels of free fatty acids or other lipids can lead to lipotoxicity, and lipid droplets help mitigate this by sequestering toxic lipids, thereby reducing cellular toxicity [154]. Notably, lipid droplets have been suggested as fundamental elements for maintaining stemness, promoting chemoresistance, and contributing to disease relapse in cancer [155, 156]. Thus, lipid droplet abundance has been shown to correlate with cancer stemness in both breast [157] and colorectal cancer [158], suggesting a functional link between lipid metabolism and the maintenance of stem‐like properties. Moreover, elevated lipid droplet accumulation has been implicated in promoting resistance to chemotherapy in prostate, breast, and colorectal cancer models, highlighting their potential role in supporting treatment persistence and tumor relapse [159, 160].

### 5.3 Autophagy, Cell Size Control, and Structural Adaptation in PGCCs

Autophagy is also a key mediator of cell size control, working in concert with growth signaling pathways to match cell size to environmental conditions and metabolic needs [161, 162, 163]. Notably, the mevalonate pathway, primarily recognized for its role in cholesterol synthesis, also regulates cell size and maintains protein homeostasis through its modulation of autophagy [163]. It has been shown to control basal autophagic flux in mammalian cells, and inhibition of this pathway leads to increased cell size and protein density. The effects of the mevalonate pathway on cell size and protein density are mediated by the geranylgeranylation of the small GTPase RAB11, which is essential for maintaining basal autophagic flux [163] (Figure 3). Further research has recently uncovered more significant connections between autophagy and the regulation of cell size. Hence, the cytoskeletal protein vimentin has been demonstrated to couple cell size and autophagy by modulating the amino acid‐sensing activity of Rag GTPase within the mTORC1 signaling pathway [162] (Figure 3). Remarkably, PGCCs exhibit elevated levels of vimentin gene expression and have a strong dependence on their vimentin network to maintain structural integrity and cellular function [164, 165]. This increased dependence strengthens the role of vimentin in coupling cell size and autophagy, serving as a key factor in the adaptability and stability of PGCCs within TME.

### 5.4 PI3P Signaling and Autophagy Membrane Dynamics in PGCCs

Phosphatidylinositol‐3‐phosphate (PI3P) plays a crucial role in regulating autophagy membrane dynamics by facilitating the formation and maturation of autophagosomes. This lipid acts as a signaling molecule that orchestrates various membrane events during autophagy [166]. Remarkably, the regulation of PI3P pools by phosphatidylinositol 5‐phosphate 4‐kinase has been shown to play a significant role in controlling both autophagy and cell size [167]. In the context of PGCCs, which display extreme polyploidy and enlarged cellular architecture, finely tuned PI3P signaling may be essential not only for maintaining efficient autophagic flux but also for coordinating intracellular membrane trafficking and organelle distribution [168]. Therefore, given the unique characteristics of PGCCs, the dynamic regulation of PI3P levels may not only support their autophagic adaptation but also regulate cellular size, enhance metabolic flexibility, and promote resilience under stress, contributing to the overall adaptability of PGCCs (Figure 3).

### 5.5 Metabolic Reprogramming and Autophagy as Mediators of PGCC Immune Resistance

PGCCs evade immune control through multiple complementary mechanisms, including altered antigen‐presentation and upregulation of immune‐checkpoint or “don't‐eat‐me” signals such as PD‐L1 and CD47 [169, 170], and an immunomodulatory secretome and extracellular vesicle output that can recruit suppressive myeloid cells and blunt effector responses [18, 46]. Finally, large‐scale genomic alterations and polyploidy themselves correlate with immune exclusion programs across cancers, providing a conceptual link between PGCC genomic state and immune evasion in situ [171, 172].

Importantly, metabolic reprogramming and autophagy may also contribute to immune escape in PGCCs by both shaping an immunosuppressive tumor microenvironment and modulating antigen presentation. Hence, the reliance of PGCCs on glycolysis and the resulting lactate production can create a local immunosuppressive microenvironment, with lactate accumulation both impairing cytotoxic T‑cell function and promoting regulatory phenotypes in myeloid cells, thereby aiding PGCC survival and immune evasion [53, 74]. On the other hand, hypoxia has been shown to downregulate MHC class I expression and reduce antigen presentation in cancer cells via the activation of autophagy and lysosomal degradation of MHC‑I and a decreased immunopeptidome, correlating with exclusion of CD8^+^ T cells from tumors [173, 174]. In fact, inhibition of autophagy under hypoxic conditions restores MHC‑I surface expression and the antigen repertoire presented to T cells, demonstrating a direct role of hypoxia‑induced autophagy in immune escape [173]. This suggests that similar autophagy‑dependent mechanisms could contribute to aberrant antigen processing and downregulation of tumor antigen presentation in PGCCs, intensifying their ability to persist under immune surveillance.

Overall, PGCC dormancy depends on a coordinated network of metabolic and autophagic adaptations. Through the integration of glycolysis, oxidative phosphorylation, lipid metabolism, and autophagy‐mediated cell size control, PGCCs sustain long‐term survival, evade immune surveillance, and retain the capacity to re‐enter the cell cycle and generate progeny.

## 6 Role of Metabolism and Autophagy in PGCC Progeny Formation

The generation of PGCC progeny represents a pivotal phase in tumor progression, linking the metabolic and autophagic adaptations of dormant PGCCs to their capacity to re‐enter proliferation. These adaptations also regulate membrane remodeling, which is essential for neosis and the budding of daughter cells. This section examines how lipid metabolism, autophagy modulation, and structural regulators coordinate to drive progeny formation and tumor relapse.

### 6.1 Lipid Metabolism as a Driver of PGCC Progeny Formation

**FIGURE 4:**
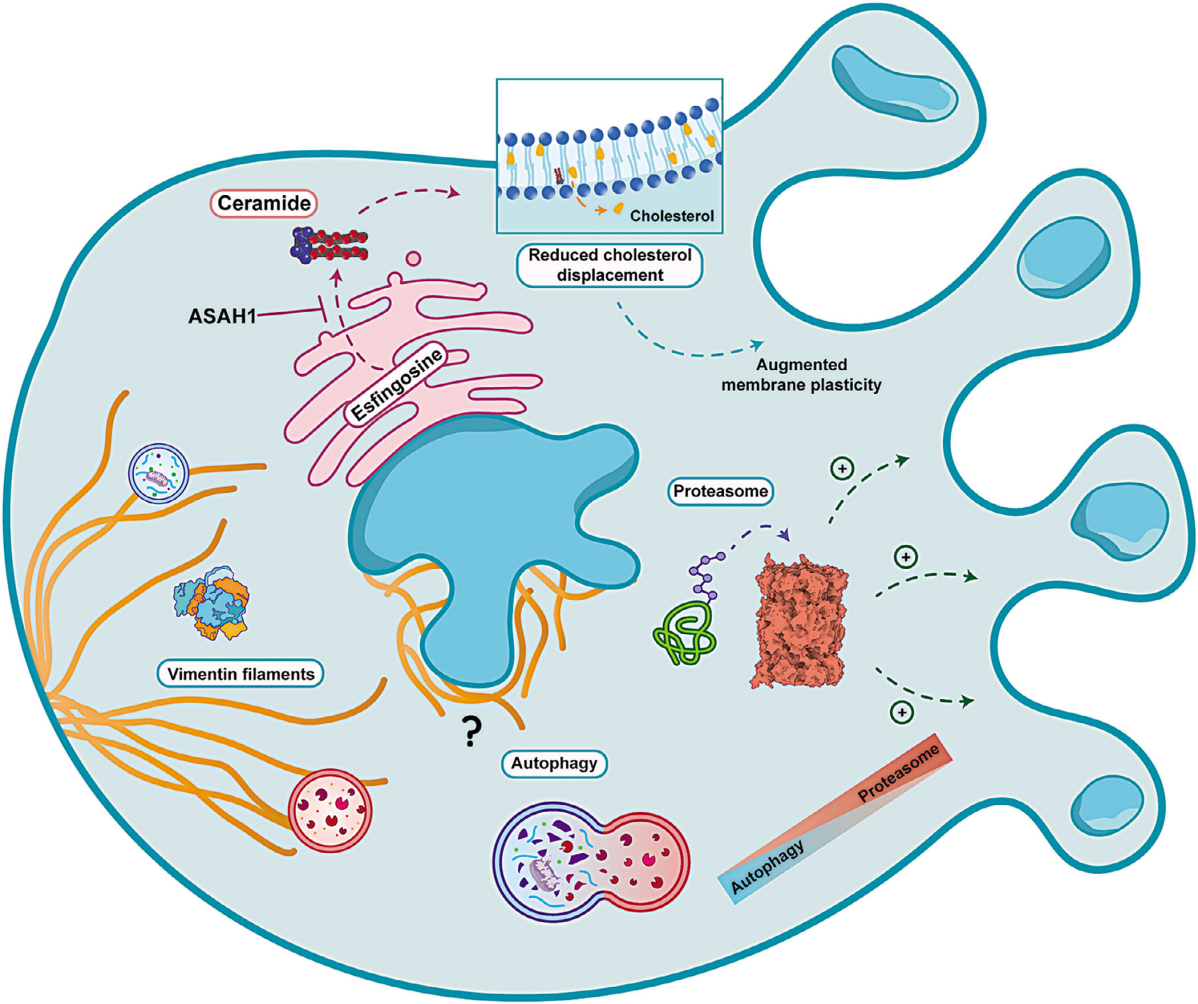
Role of metabolism and autophagy in PGCC progeny formation. As ceramide displaces cholesterol from the plasma membrane, acid ceramidase (ASAH1) promotes neosis by reducing ceramide levels, which enhances membrane fluidity and enables PGCCs to generate progeny through budding. Additionally, the suppression of autophagy may be necessary for the effective involvement of ubiquitin‐mediated mechanisms in cell membrane remodeling. The “?” symbol in the figure denotes a proposed role, referring to the possibility that, besides its structural function in mTORC1 signaling and autophagy regulation, vimentin may also be involved in nuclear fragmentation and micronuclei formation required for the reductive depolyploidization of PGCCs. These interconnected processes enable PGCCs to produce smaller, proliferative daughter cells, contributing to tumor progression and therapy resistance. See text for details.

Importantly, recent research has shown that the adjustment of metabolic and autophagic pathways also plays a crucial role in the formation of PGCC progeny, thereby contributing to cancer relapse (Figure 4). In particular, lipid metabolism has emerged as a crucial factor in the formation of PGCC progeny. In this context, the inhibition of acid ceramidase (ASAH1), which is an enzyme that hydrolyzes ceramide into sphingosine and fatty acid, has been shown to prevent asymmetric cell division, particularly during neosis. Importantly, while ASAH1 is upregulated in PGCCs, it is not necessary for their formation; however, ASAH1 is essential for the process of PGCC neosis [175]. In a subsequent study, it was demonstrated that ceramide synthase 6, which preferentially generates C16‐ceramide, enhances the ability of the tumor suppressor p53 to inhibit progeny formation in PGCCs. This finding suggests that reducing levels of C16‐ceramide is crucial for facilitating the formation of PGCC progeny [176]. Moreover, PGCCs have been shown to be highly dependent on cholesterol for progeny formation through amitotic division [177]. As ceramide appears to displace cholesterol from the plasma membrane, lowering ceramide levels may increase membrane fluidity, facilitating the capacity of PGCCs to generate progeny through budding (Figure 4) [177]. Ceramide also influences key signaling pathways involved in cell fate decisions by modulating the activity of Akt and PP2A [178], as well as altering the lipid raft localization of critical growth factor receptors such as EGFR and IGF1R [179]. These effects on membrane organization and signal transduction may have important implications for the formation and behavior of progeny cells emerging from PGCCs [180], potentially influencing their survival, proliferation, and therapeutic resistance.

### 6.2 Autophagy and Ubiquitin‐Mediated Membrane Remodeling in PGCC Progeny Formation

As discussed above, the induction of autophagy appears to be necessary for the formation of PGCCs [125]. In contrast, the inhibition of autophagy may facilitate the generation of progeny from these cells [126]. The induction of autophagy for the initial formation of PGCCs may be essential for providing the cellular resources needed to survive stress and support their development. However, it is not immediately clear why the suppression of autophagy might facilitate the generation of progeny from PGCCs. Studies on lipid metabolism suggest that cell membrane remodeling may be essential to the budding process by which daughter cells are generated from PGCCs. It is notable that ubiquitin and ubiquitin‐like proteins can directly associate with proteins and lipids, influencing membrane curvature and dynamics [181]. There is a compensatory balance between autophagy and other ubiquitin‐mediated processes, such as the ubiquitin‐proteasome system; inhibition of one leads to the upregulation of the other [182]. In the context of PGCC progeny formation, it is conceivable that the suppression of autophagy may be necessary for the effective involvement of ubiquitin‐mediated mechanisms in cell membrane remodeling (Figure 4). Hence, suppression of autophagy in PGCCs may facilitate progeny formation by redirecting ubiquitin‐mediated processes toward membrane remodeling rather than degradation. Ubiquitin and ubiquitin‐like modifiers can tag membrane‐associated proteins or lipids, promoting the recruitment of curvature‐inducing scaffolds such as ESCRT complexes and BAR‐domain proteins, which drive membrane invagination, scission, and budding [183, 184]. When autophagy is inhibited, substrates that would normally be targeted for lysosomal degradation may instead engage these ubiquitin‐dependent trafficking pathways, enhancing membrane remodeling necessary for the budding of daughter cells from PGCCs. This provides a mechanistic link between autophagy suppression and the structural reorganization of membranes that underlies PGCC progeny generation.

### 6.3 Vimentin as a Structural and Autophagic Regulator in PGCCs

Vimentin is a type of intermediate filament protein that plays a crucial role in maintaining the structural integrity and stability of cells [185]. High vimentin expression is associated with tumor aggressiveness, metastasis, and poor prognosis, and it is considered a hallmark of mesenchymal‐like cancers, particularly in processes like EMT, where it contributes to the invasiveness of tumors [186]. Remarkably, vimentin intermediate filaments are essential for maintaining the structural integrity of PGCCs [164], and their nuclear localization has been linked to the enhanced migration and invasion capacity of daughter cells derived from PGCCs [187]. Moreover, recent findings indicate that vimentin plays a role in the nuclear segmentation of granulocytes [188], suggesting that this intermediate filament protein may also play a significant role in the nuclear fragmentation and micronuclei formation necessary for the reductive depolyplodization of PGCCs (Figure 4). Additionally, vimentin plays a key role in regulating the cytoplasmic positioning of autophagosomes and lysosomes, making it a crucial factor in the control of mTORC1 activity and autophagy [189]. This suggests that the unique structural demands of PGCCs may likewise influence their autophagic capacity in a significant way (Figure 4).

Collectively, recent research supports that the successful generation of PGCC progeny relies on a finely tuned coordination of lipid metabolism, autophagy modulation, and structural support through vimentin. By orchestrating membrane dynamics, energy metabolism, and cytoskeletal integrity, PGCCs can efficiently produce daughter cells, providing a mechanistic bridge to understanding how metabolic and autophagic processes influence tumor growth and relapse.

## 7 Therapeutic Targeting of PGCCs

Despite compelling evidence supporting PGCCs as key drivers of therapy resistance and tumor relapse, their clinical exploitation remains largely unexplored, with no clinical trials specifically designed to target this cell population. Nevertheless, a growing body of preclinical studies has begun to identify specific vulnerabilities of PGCCs and to propose targeted strategies aimed at impairing their formation, survival, and tumor‐repopulating capacity (Table 1). Although these approaches have not been developed with PGCCs as a primary target, they provide an important clinical framework for their potential therapeutic exploitation.

**TABLE 1:** Preclinical studies targeting PGCC vulnerabilities.

Cancer type	Targeted pathway	Therapeutic approach	Model	Ref.
Breast cancer	Metabolism reprogramming (ferroptosis sensitivity)	Single‐cell multiomics screening	Cell lines	[58]
	PGCC formation	Ganciclovir	Cell lines	[199]
Bladder cancer	Metabolism reprogramming (lipid/mitochondrial metabolism, ROS)	Zoledronic acid	Cell lines	[191]
Ovarian cancer	Autophagy regulation (PGCC progeny formation)	Autophagy modulation (hydroxychloroquine, nelfinavir)	Cell lines	[126]
	mTOR, HR, and DNA checkpoint pathways	mTOR + PARP inhibitors	Cell lines/human specimens	[193]
	PGCC formation and tumor growth (IL‐6 blocking)	Tocilizumab/Apigenin	Cell lines/xenografts	[201]
Ovarian/Breast cancer	Cell cycle/endoreplication (PGCC formation and depolypoidization)	Mifepristone + Olaparib	Cell lines, organoids, xenografts	[195]
Prostate cancer	Sphingolipid metabolism (ASAH1; neosis)	ASAH1 inhibition/C_16_‐ceramide modulators	Cell lines	[175, 176]
Glioblastoma	Cell cycle (proliferation and clonogenic formation)	PLK1 inhibitors	Cell lines	[194]
Colorectal/Breast cancer	Epigenetic regulation/transcriptional plasticity	HDAC inhibitors	Cell lines	[200]
Non–small cell lung cancer cells	Cell cycle, PGCC formation (IL‐1B inhibition)	IL‐1β inhibitors	Cell lines	[202]
Nasopharyngeal carcinoma	PGCC dormancy, autophagy inhibition	Autophagy inhibitors (BML‐275)	Cell lines/xenografts/human specimens	[125]
Colorectal cancer/Glioblastoma/Astrocytoma	Metabolism (mitochondrial fission)	T‐401 (microtubule assembly inhibitor)	Cell lines	[192]
Gastric/ovarian cancer/melanoma	PGCCs survival	PRL3‐zumab (PRL3 block)	Cell lines, xenografts	[203]

Abbreviations: ASAH1, acid ceramidase; HR, homologous recombination; IL‐1β, interleukin‐1 beta; IL‐6, interleukin‐6; PARP, poly(ADP‐ribose) polymerase; PLK1, polo‐like kinase 1; PRL3, phosphatase of regenerating liver 3; ROS, reactive oxygen species.

### 7.1 Targeting Metabolic Reprogramming and Mitochondrial Function

A major axis of targeting involves metabolic reprogramming, as PGCCs display profound alterations in lipid metabolism, mitochondrial function, and redox homeostasis. Consistent with this, single‐cell multi‐omics approaches have identified metabolic vulnerabilities, including ferroptosis sensitivity in breast PGCCs [58]. In addition, zoledronic acid, a potent bisphosphonate drug used to treat osteoporosis by targeting osteoclasts, the cells responsible for bone resorption [190], has been shown to disrupt lipid and mitochondrial metabolism, leading to selective impairment of cisplatin‐induced prostate PGCC survival [191]. More recently, targeting mitochondrial dynamics and cellular energy homeostasis has emerged as a promising strategy, as demonstrated by the microtubule assembly inhibitor ST‐401, which induces a transient integrated stress response, reduces energy metabolism, and promotes mitochondrial fission, ultimately impairing PGCC development [192].

### 7.2 Autophagy as a Therapeutic Vulnerability

In addition to metabolic reprogramming, autophagy represents a central vulnerability in PGCC biology, acting as a key regulator of cellular adaptation to stress. In particular, pharmacological inhibition of autophagy has been reported to impair the ability of PGCCs to generate proliferative progeny, thereby limiting tumor repopulation capacity. Consistently, autophagy‐targeting strategies, including the use of clinically available inhibitors such as hydroxychloroquine, nelfinavir, and/or rapamycin, have demonstrated efficacy in reducing PGCC‐derived colony formation in ovarian cancer models [126]. You et al. [125] have recently provided evidence indicating that pretreatment with an autophagy inhibitor before cisplatin‐based chemotherapy can prevent the formation of cisplatin‐induced PGCCs in nasopharyngeal carcinoma, thereby reducing the risk of recurrence and metastasis. In this framework, mTOR signaling has been identified as a central regulatory pathway that can be therapeutically exploited, as mTOR inhibition not only disrupts autophagy‐related processes but also modulates DNA damage response pathways, leading to reduced PGCC burden and enhanced sensitivity to poly (ADP‐ribose) polymerase (PARP) inhibitors in CCNE1‐amplified ovarian cancer models [193].

### 7.3 Targeting PGCC Progeny Generation Mechanisms

Beyond these vulnerabilities, targeting the mechanisms underlying PGCC progeny generation represents a particularly promising approach. Accordingly, interference with these processes has been shown to effectively impair tumor regeneration. For instance, inhibition of ASAH1, a key regulator of sphingolipid metabolism, selectively blocks PGCC progeny formation without affecting their initial formation, highlighting a stage‐specific vulnerability [175]. Consistently, additional studies have demonstrated that the modulation of C16‐ceramide levels, either through ceramide synthase 6 or p53, can effectively impair the generation of proliferative daughter cells from PGCCs, further reinforcing that sphingolipid metabolism is of functional importance in these cells and has potential for clinical intervention [176]. On the other hand, targeting mitotic regulators such as polo‐like kinase 1 (PLK1) induces mitotic arrest and reduces clonogenic capacity, further supporting the therapeutic potential of disrupting cell cycle dynamics in PGCC‐driven tumor regrowth [194].

### 7.4 Therapeutic Repurposing of Contraceptive Agents in PGCC Targeting

Due to their developmental similarities with blastomere‐stage embryos, PGCCs have been proposed as representing “somatic blastomeres” [53, 148]. Accordingly, it has been suggested that contraceptive drugs could serve as potential therapies against PGCCs. In this context, a recent study has shown that the combination of mifepristone, an emergency contraceptive, and the PARP inhibitor olaparib significantly suppressed the development of olaparib‐induced PGCCs in ovarian cancer cells [195]. Mifepristone synergizes with olaparib to induce apoptosis in cells undergoing endoreplication, effectively preventing the formation of new PGCCs, especially those induced by therapeutic stress. The known contraceptive mechanisms of mifepristone are primarily mediated by its antagonist effects on progesterone and glucocorticoid receptors [196]. However, mifepristone has also been shown to affect cellular metabolism and autophagy. Hence, mifepristone enhances insulin‐stimulated glucose uptake in skeletal muscle by impairing mitochondrial function and promoting the activation of AMPK [197]. Additionally, mifepristone has been reported to induce autophagy in polycystic ovarian condition models [198]. Therefore, mifepristone may exploit the metabolic and autophagy dependencies of PGCCs, hindering their adaptation to stress and promoting their elimination, especially when combined with therapies like olaparib.

### 7.5 Epigenetic, Inflammatory, and Oncogenic Vulnerabilities in PGCCs

Epigenetic regulation represents an additional layer of vulnerability, as the pronounced transcriptional plasticity of PGCCs is tightly controlled by chromatin remodeling mechanisms. Based on this, EZH2‐driven epigenetic reprogramming has been linked to PGCC formation and stemness features, particularly in the setting of oncogenic viral signaling (HCMV), further supporting the potential of targeting epigenetic regulators such as EZH2 or HDAC inhibitors [199, 200].

Furthermore, inflammatory signaling pathways also contribute to PGCC biology. For instance, cytokine‐mediated feedback loops involving IL‐6 have been shown to promote PGCC formation, stemness, and tumor microenvironment remodeling, including the induction of cancer‐associated fibroblasts [201]. Similarly, IL‐1β has been implicated in the regulation of PGCC‐associated senescence and drug resistance, highlighting the role of inflammatory mediators as potential therapeutic targets [202]. Moreover, emerging studies have identified novel therapeutic targets associated with PGCC survival and relapse. For example, phosphatase of regenerating liver 3 (PRL3) overexpression has been linked to PGCC formation and resistance to genotoxic stress, and targeting this pathway with specific antibodies such as PRL3‐zumab has shown promise in reducing tumor relapse in preclinical models [203].

### 7.6 Translational Challenges

Collectively, these studies highlight that PGCCs can be therapeutically exploited through multiple, stage‐specific vulnerabilities. Notably, many of these strategies do not necessarily aim to eliminate PGCCs directly, but rather to impair their ability to survive under stress or to regenerate tumor cell populations. This distinction underscores the importance of understanding the dynamic life cycle of PGCCs when designing therapeutic interventions. However, despite promising preclinical evidence, the translation of these approaches into clinical applications remains limited, reflecting significant challenges in the identification, targeting, and functional characterization of PGCCs in patient settings.

## 8 Emerging Technologies Transforming PGCC Research

Due to their complex nature, including low abundance, high heterogeneity, and dynamic behavior, the study of PGCCs has been historically hampered by conventional approaches. Traditionally, PGCCs detection has been based on morphology, ploidy, and cell‐cycle markers, using microscopy, cytological staining, and flow cytometry [47, 204]. In recent years, instead, innovative technologies have emerged that not only overcome these limitations but also enable more precise characterization of PGCCs and open new avenues for the development of targeted therapies against this cellular population, providing unprecedented insights. The main technological advances, along with their specific goals, advantages, and limitations, are summarized in Table 2.

**TABLE 2:** Emerging technologies for PGCC research.

Technology	Specific technology	Advantages	Limitations	Ref.
Single‐cell and spatial multi‐omics	Multimodal and single‐cell technologies	Simultaneous interrogation of multiple molecular layers	Lacks spatial context; technically challenging; high‐cost	[58]
	Spatial transcriptomics	Spatial context	Technically challenging, high‐cost, and not yet standardized for PGCC research	[47]
	ISET (Isolation by size of epithelial tumor cells)	Non invasive	Requires immunomorphological validation to exclude non‐PGCCs	[205]
Live‐cell imaging and lineage tracing	Time‐lapse systems & fluorescent labeling (e.g., FUCCI, CK‐GFP)	Captures real‐time processes	Data‐intensive; stable labelling or barcodes are required	[206, 207]
	Multispectral imaging flow cytometry (MIFC)	High‐resolution spatial and temporal analysis; reveals information about rare events	Requires specialized equipment; lacks transcriptomic readout; high‐cost	[208]
Preclinical models	Tumor spheroids	Affordable, relatively easy to generate; more physiologically relevant than 2D cultures	Lacks a full tumor microenvironment	[58]
	Patient‐derived organoids (PDOs)	Preserves tumor heterogeneity	High variability between samples and time‐consuming; still lacks a full in vivo architecture	[195, 207]
	In vivo models (PDXs and xenografts)	Preserves tumor heterogeneity and reflects complex in vivo responses	High complexity and variability; longitudinal tracking can be challenging	[148, 180, 195]
	Co‐cultures	Recapitulates tumor‐stroma and tumor‐immune interactions; more physiologically relevant than monocultures	High complexity and variability; still lacks a full in vivo architecture	[201]
Computational models and systems biology	Bioinformatic tools and databases	Robust integration of large, complex datasets into altered functional pathways; transforms raw genomic data into meaningful biological insights	Data‐intensive and need for experimental validation	[60]
	Interactive Web Portals (e.g., PGCC Explorer)	Efficient screening of compounds to target PGCCs	New compounds and additional cell lines are required	[210]
	Deep learning and advanced microscopy (CellFDet, OCDet)	Fast analysis of a batch with multiple preparations	Requires specialized and exhaustive training	[212, 213]
	Mathematical modelling	Predicts adaptive trajectories and links karyotypic adaptation with drug sensitivity in PGCCs	Can rely on assumptions regarding cellular homogeneity; need experimental validation	[215, 216]

Abbreviations: CK‐GFP, cytokeratin‐green fluorescent protein; FUCCI, fluorescent ubiquitination‐based cell cycle indicator; MIFC, multispectral imaging flow cytometry; PDOs, patient‐derived organoids; PDXs, patient‐derived xenografts.

### 8.1 Single‐Cell and Spatial Multi‐Omics

Among these advances, combining single‐cell RNA sequencing (scRNA‐seq) with various approaches has yielded new insights into PGCCs. However, this study relies on a single molecular layer (transcriptomics) and does not fully capture the regulatory mechanisms underlying cell state transitions. To solve this problem, more comprehensive multimodal single‐cell technologies have emerged, enabling the simultaneous interrogation of multiple molecular layers within the same cell. Building on this, Zhou et al. combined scRNA‐seq with high‐content imaging and functional assays, linking transcriptional states to PGCC morphology and identifying PGCC‐specific vulnerabilities [58]. Despite these advances, current approaches still lack spatially resolved molecular information, limiting our understanding of PGCC organization within the tumor context. Spatial transcriptomics may help address this limitation by preserving tissue architecture while enabling gene expression profiling. Nevertheless, these strategies remain technically challenging, costly, and not yet fully standardized, particularly for PGCCs [47]. On the other hand, recent advancements in liquid biopsy have enabled the detection of circulating PGCCs. The isolation by size of tumor cells (ISET) technique allows the detection of circulating PGCCs in blood, using a membrane with 8 µM diameter‐multipores [205]. Then, membranes with PGCCs can be fixed and stained for further analysis, offering a promising tool for the clinical monitoring and prognostic assessment of PGCCs in liquid biopsies.

In summary, these complementary technologies provide a framework to link PGCC molecular and spatial heterogeneity with their clinical relevance in tumor progression and liquid biopsy detection.

### 8.2 Live‐Cell Imaging and Lineage Tracing of PGCCs

Complementary imaging approaches, including time‐lapse systems combined with lineage tracking strategies, are able to capture dynamic processes over time, rather than a static snapshot. Particularly, Song et al. [206] observed that mitochondria, the Golgi apparatus, and nuclear structures are asymmetrically inherited among PGCC daughter cells, highlighting heterogeneity in both morphology and dynamic behavior. Additionally, they demonstrated that PGCCs displayed prolonged G1/S transitions, asynchronous micronuclei, and intranuclear mitosis using the FUCCI system [206]. Other approaches are based on the use of CK‐GFP to visualize the cell membrane and H2B‐mCherry to label the nucleus, allowing the dynamic tracking of cellular and nuclear morphology over time [207]. Besides, multispectral imaging flow cytometry (MIFC) has emerged as an innovative bridging technology, combining the analytical capacity of conventional flow cytometry with the spatial resolution of microscopy. In connection with the PGCCs, MIFC has been successfully used to identify PGCCs and characterize their associated senescence program due to CD147 depletion by acquiring multispectral images of individual PGCCs [208].

Collectively, these imaging strategies enable the integration of spatial and temporal information to dissect the complex dynamic behavior and molecular heterogeneity of PGCCs.

### 8.3 Organoid and Xenograft Models

Conventional 2D culture of PGCCs is based on the administration of inducers such as paclitaxel [57], CoCl_2_ [19], doxorubicin [14], or radiation [22], among others. These agents selectively kill common diploid tumoral cells and simultaneously promote the transformation of the surviving cells into PGCCs via endoreduplication or cell fusion [169]. Despite its utility, 2D culture models present important limitations, since they do not recapitulate the spatial organization, mechanical constraints, and microenvironmental gradients properly. In contrast, preclinical models such as spheroids, patient‐derived xenografts (PDXs), and patient‐derived organoids (PDOs) are able to mimic the natural 3D tumor architecture.

In the case of tumor spheroids, these structures can be generated using approaches such as microfluidic systems, which establish a nutrient and hypoxic gradient that enhances PGCCs generation. Spheroids provide a suitable platform to investigate stem‐like properties of PGCCs and are particularly useful for drug screening [58]. On the other hand, PDOs represent a suitable approach for 3D culture of PGCCs because preserves better tumor heterogeneity in comparison to tumor spheroids. For example, high‐grade serous carcinoma‐derived organoids have been established to capture the spatiotemporal dynamics of PGCC formation and fate [207]. Moreover, PDOs and PDXs have revealed a link between PGCCs and PARP inhibitor resistance, where their targeting improves Olaparib efficacy in ovarian cancer models [195]. In other in vivo studies, both PDXs and xenografts were used as a powerful platform to evaluate the effect of disrupting lipid raft microdomains, demonstrating that their inhibition impairs PGCC budding, reduces tumor repopulation, and overcomes the resistance to radiotherapy [180], while their daughter cells exhibit mesenchymal traits in immunocompromised mice, highlighting their plasticity [148]. Despite their relevance, PDXs and PDOs offer limited experimental flexibility to dissect cell‐cell interactions within the tumor microenvironment. In this regard, cocultures facilitate the study of interactions with stromal and immune cells, including mechanisms of immune evasion and tumor reprogramming [201].

Altogether, these preclinical models provide a versatile experimental framework to investigate PGCC biology, encompassing both tumor‐intrinsic properties and microenvironmental interactions.

### 8.4 Computational Models and Systems Biology Frameworks

The increasing use of advanced experimental techniques has led to the generation of large and complex datasets, highlighting the need for robust bioinformatic tools for their integration and analysis. In this context, the development of mathematical and computational models to study PGCCs is a promising area in cancer research.

Currently, researchers increasingly rely on public data repositories such as the Gene Expression Omnibus (GEO) and the Kyoto Encyclopedia of Genes and Genomes (KEGG), which provide access to high‐throughput gene expression datasets. This data is often complemented with Whole Exome Sequencing (WES) and bioinformatics tools, such as DAVID, GO, KEGG, and GSEA, that can translate differential gene expression into altered functional pathways. In addition, protein–protein interactions can be explored using other tools, including MCODE, STRING, and Cytoscape [209]. In line with this analytical framework, recent studies have begun to apply integrative strategies to specific biological questions. For example, one study combined transcriptomic profiling with protein–protein interaction network‐level interpretation to characterize the macroevolutionary patterns of PGCCs during their transition back to a multicellular life cycle [60].

In the realm of pharmacogenomics, platforms like the interactive web portal PGCC Explorer have been developed for high‐throughput drug screening. This tool integrates the efficacy of thousands of compounds to efficiently target PGCCs from six different cell lines, identifying novel biomarkers and related vulnerabilities. Additionally, this platform includes genomic information such as mutations, copy number alterations, gene expression, and pathway activities [210].

As mentioned above, PGCCs were usually identified by pathologists in tissue preparations, a process that relied on specialized expertise and was labor‐intensive. Additionally, there is heterogeneity regarding PGCC morphology [211]. Advanced microscopy paired with deep learning algorithms addresses these challenges, enabling high‐throughput quantification of PGCC number, size, shape, multinucleation, and subcellular structures [212, 213]. Many of these algorithms, such as CellFDet and OCDet, are trained to recognize and classify cells directly in histological images based on features such as extreme size, multinucleation, nuclear atypia, hyperchromatism, and nucleo‐cytoplasmic ratio. Interestingly, OCDet is also capable of detecting immune cells as well [212], while CellFDet also recognizes mitotic figures, evaluating cellular proliferation activity and the genomic instability of the tumor [213].

Finally, mathematical oncology integrates diverse modeling frameworks to describe tumor dynamics and treatment adaptation. Population‐based, stochastic, fitness landscape, and evolutionary models have been extensively discussed in the literature to account for tumor heterogeneity [214]. More recent state‐structured approaches explicitly incorporate phenotypic plasticity, including polyaneuploid cancer cell (PACC)‐like states associated with PGCC biology [215]. Additionally, at a more mechanistic level, the CLONEID framework can predict how metabolic stress and karyotype dynamics sculpt drug sensitivity over time [216].

Overall, these approaches highlight the value of integrative modeling to unravel the complexity of polyploid tumor evolution, while capturing the dynamic and stress‐adaptive nature of PGCC states.

## 9 Challenges, Knowledge Gaps, and Future Directions

Despite the growing recognition of PGCCs as key players in tumor evolution, metastasis, and therapy resistance, their study remains hindered by several conceptual and technical challenges that limit both mechanistic understanding and clinical translation. One of the most significant obstacles lies in the difficulty of isolating and maintaining PGCCs in vitro, as these cells are often transient, heterogeneous, and highly plastic, with no universally accepted biomarkers or high‑throughput isolation methods currently available [204, 217]. In this context, flow cytometry methods based on DNA content are limited and drastically reduce viability [40]. Furthermore, research is complicated by variable definitions and culture conditions across studies, including inconsistent size thresholds and stress induction protocols, which affect PGCC induction and characterization reproducibility [204]. Importantly, conventional cell culture systems are poorly suited to recapitulate the dynamic tumor microenvironment that supports PGCC survival and function. For instance, none of the standard culture conditions accurately replicate the environment experienced by cancer cells within a primary tumor, including the biomechanical stress induced by the tumoral matrix, in the generation of PGCCs [218]. Current models still fail to capture the full diversity and dynamics of PGCCs, and future research must integrate microfluidics, patient‐derived materials, and advanced imaging to better understand PGCC biology and develop targeted therapies.

A closely related limitation is the lack of specific and universally accepted markers for PGCCs, which complicates their identification, isolation, and quantification across experimental models and patient samples. While morphological criteria such as increased cell size and multinucleation are commonly used, these features are not exclusive to PGCCs and may overlap with other cell states, including senescence or failed cytokinesis. At the molecular level, PGCCs frequently express stemness‐associated factors such as OCT4, NANOG, and SOX2, as well as markers linked to stress responses and DNA damage; however, none of these are specific enough to uniquely define this population [148, 204]. This lack of specificity has led to inconsistencies in the field and underscores the urgent need for robust molecular signatures or combinatorial marker panels that can reliably distinguish PGCCs from other tumor cell states.

Another major gap concerns the limited availability of in vivo lineage‐tracing approaches capable of definitively demonstrating the origin, fate, and functional contribution of PGCCs during tumor progression. While in vitro studies and indirect evidence support the idea that PGCCs can generate proliferative progeny through neosis and contribute to tumor repopulation, direct in vivo evidence remains scarce. Advanced genetic labeling strategies [219], single‐cell lineage‐tracing technologies [220], and intravital imaging approaches [221] will be essential to track PGCC dynamics within tumors and to establish their causal role in disease progression and metastasis.

Finally, a major challenge in PGCC research is clarifying the contribution of these cells to clinical recurrence and patient outcomes. Although PGCCs are frequently observed in high‐grade tumors and following treatment, and their presence correlates with aggressive phenotypes and poor prognosis in several cancer types [170, 222, 223], definitive clinical evidence demonstrating that PGCCs directly drive relapse remains incomplete. This gap is partly due to the lack of standardized detection methods and longitudinal studies that monitor PGCC dynamics in patient samples over time. Addressing this issue will require integrating histopathological analyses with molecular profiling and clinical follow‐up data to establish clear associations between PGCC abundance, therapeutic response, and disease recurrAenceU.

## 10 Conclusions and Prospects

The challenge of drug resistance and cancer recurrence remains a significant hurdle in oncology. The phenomenon of tumor dormancy, particularly in the context of PGCCs, underscores the complexity of cancer biology. PGCCs, once thought to be merely nondividing cells, are now recognized for their potential to produce proliferative progeny, contributing to relapse and treatment resistance.

In this review, we have highlighted recent evidence demonstrating that the ability of PGCCs to undergo metabolic adaptation and modulate autophagy represents a central feature supporting all stages of their life cycle, including formation, long‐term dormancy and survival, and progeny generation. Conventional anticancer treatments are largely designed to eliminate rapidly proliferating cells, whereas PGCCs often reside in a dormant or slow‐cycling state that renders them intrinsically resistant. Moreover, their ability to undergo depolyploidization and regenerate heterogeneous tumor cell populations further complicates treatment outcomes. From a therapeutic perspective, targeting the unique vulnerabilities of PGCCs, particularly their altered metabolic and autophagic states, represents a promising but still largely underexplored avenue for intervention.

Looking forward, several key research priorities emerge for the field. These include the development of improved in vitro and in vivo models that faithfully capture PGCC biology, the identification of specific molecular markers and regulatory pathways, and the integration of multi‐omics approaches to define the epigenetic and transcriptional landscapes of PGCCs at high resolution. Additionally, advancing single‐cell and spatial transcriptomics technologies will be critical to dissect the heterogeneity and microenvironmental interactions of PGCCs within tumors. Finally, the clinical translation of PGCC research will require not only the development of agents specifically targeting these cells, but also their successful progression from bench to bedside, including the design of appropriate clinical trials, the establishment of patient selection criteria, and the identification of reliable biomarkers to evaluate treatment efficacy and safety. By addressing the giant dormant cancer cell challenge, we may contribute to the development of more effective therapeutic strategies and ultimately improve outcomes for patients facing cancer relapse.

## Author Contributions

**S.G.‐L., M.T.S.‐M**. writing – original draft. **A.D.‐C**. visualization. **A.R.‐A**. conceptualization, writing – review and editing. All authors have read and approved the final manuscript.

## Funding

This work was supported by Ministerio de Ciencia e Innovación, Agencia Estatal de Investigación, Spain (PID2019‐105256RB‐I00/AEI/10.13039/501100011033), and Instituto de Salud Carlos III (ISCIII), Spain (PI23/00489s), co‐funded by the European Union. A.R.‐A. was funded with a researcher contract through the program ‘Nicolás Monardes’ from Junta de Andalucía. M.T.S‐M. was funded by a predoctoral researcher contract from Ministerio de Ciencia e Innovación, co‐funded by the European Social Fund. The funding sources are neither involved in the writing of this review nor in the decision to submit the article for publication.

## Ethics Statement

The authors have nothing to report.

## Conflicts of Interest

The authors declare no conflicts of interest.

## Data Availability

The authors have nothing to report.
